# Assessment of Suspected Breast Lesions in Early-Stage Triple-Negative Breast Cancer during Follow-Up after Breast-Conserving Surgery Using Multiparametric MRI

**DOI:** 10.1155/2022/4299920

**Published:** 2022-02-18

**Authors:** Lamiss Mohamed Abd El Aziz Sad, Naglaa Lofty Dabees, Dareen Abd El-Aziz Mohamed, Amr Tageldin, Samar Galal Younis

**Affiliations:** ^1^Clinical Oncology, Faculty of Medicine, Tanta University, Egypt; ^2^Faculty of Medicine, Tanta University, Egypt

## Abstract

**Background:**

The local recurrence rate of triple-negative breast cancer (TNBC) can be as high as 12%.The standard treatment for early-stage TNBC is breast-conserving surgery (BCS), followed by postoperative radiotherapy with or without chemotherapy. However, detection of the local recurrence of the disease after radiotherapy is a major issue.

**Objective:**

The aim of this study was at investigating the role of dynamic and functional magnetic resonance imaging (MRI) during follow-up after BCS and radiotherapy with/without chemotherapy to differentiate between locoregional recurrence and postoperative fibrosis. *Patients and Methods*. This prospective study was conducted at the oncology, radiology, and pathology departments, Tanta University. It involved 50 patients with early-stage TNBC who were treated with BCS, followed by radiotherapy with/without chemotherapy. The suspected lesions were evaluated during the follow-up period by sonomammography. All patients were subjected to MRI, including conventional sequences, diffusion-weighted imaging (DWI), and dynamic postcontrast study.

**Results:**

Ten cases were confirmed as recurrent malignant lesions. After contrast administration, they all exhibited irregular T1 hypodense lesions of variable morphology with diffusion restriction and positive enhancement. Eight cases displayed a type III curve, while two showed a type II curve. Histopathological assessment was consistent with the MRI findings in all eight cases. The combination of the data produced by DWI-MRI and dynamic contrast-enhanced (DCE) MRI resulted in 100%sensitivity, 92.5% specificity, 90.9% positive predictive value, 100% negative predictive value, and 98% accuracy.

**Conclusion:**

Combination of DWI-MRI and DCE-MRI could have high diagnostic value for evaluating postoperative changes in patients with TNBC after BCS, followed by radiotherapy with/without chemotherapy. *Trial Registrations*. No trial to be registered.

## 1. Introduction

Triple-negative breast cancer (TNBC) differs substantially from other molecular subtypes of breast cancer in terms of local recurrence, which is observed in 10–12% of patients [1, 2]. Breast-conserving surgery (BCS) involving either lumpectomy or quadrantectomy with or without axillary lymph node dissection, followed by radiotherapy with or without chemotherapy, is the standard treatment for early-stage breast cancer (stages I and II) [3].

One of the biggest challenges faced by oncologists after postoperative radiotherapy is the presence of increased density or an overt mass in mammograms that is difficult to distinguish from local recurrence [4]. In radiotherapy, the rate of local recurrence has reached nearly 3% every year. However, it is mostly detected in the first 2–3 years postsurgery [5–7]. Sensitivity of detection of local recurrence varies depending on the modalities of diagnostic radiology. For instance, for mammography and ultrasonography, the detection sensitivity is in the range of 64–71% and 81–85%, respectively [8].

Magnetic resonance imaging (MRI) of the breast after BCS is not currently recommended as routine follow-up, except in the cases of suspicious clinical or radiological evidences of local recurrence [9, 10]. To enable differentiation of breast masses, the use of the combination of dynamic contrast-enhanced (DCE) and diffusion-weighted imaging (DWI) MRI has been proposed [11, 12]. DCE-MRI has evolved as an important diagnostic tool for detecting breast-related diseases due to its capability of diagnosis, detection, and malignancy monitoring. Moreover, it is a noninvasive technique with an advantage of three-dimensional visualization, which helps to visualize the extent of the disease. It is one of the most accurate and sensitive diagnostic imaging techniques as it shows various malignant MRI features that could not be identified with the use of mammography or ultrasound [13–15]. For example, in DCE-MRI, there is a possibility of obtaining and analyzing both morphological and functional features of malignancy [16].

By the administration of a contrast agent in DCI-MRI, different enhancement techniques could be identified. Benign lesion is indicated by a slow, continuous enhancement curve (type I), and benign/malignant lesion is indicated by a medium or strong enhancement followed by a plateau (type II). Type III, which is represented by fast initial enhancement and washout, is characteristic of malignancies that occur due to increased vascular permeability and interstitial fluid [17]. Montemurro et al. have studied 75 patients of breast cancer who underwent DCE-MRI followed by core biopsy and found a statistically significant association between features of DCE-MRI and histopathological characteristics of the tumor [18].

Although a lot of excellent information regarding the morphology of tumor and limited neoangiogenesis is provided by DCE-MRI, DWI-MRI imaging was introduced for assessing additional functional information of tumor along with increased specificity while maintaining sensitivity [19]. DCE-MRI is an advanced technique of MRI capable of measuring the mobility of water molecules diffusing in the tissue, which is affected by various biophysical characteristics including the density of cell, membrane integrity, and microstructure. It has very less acquisition time and wider availability of most of the commercial scanners and does not need any contrast agent to be administered. Owing to these properties, the use of DWI has increased considerably for the detection of breast cancer. Various single-center studies have reported the importance of DWI in the diagnosis and characterization of breast cancer [20]. Since DWI-MRI could evaluate tumor response in vivo in a noninvasive manner, it could be helpful in modifying the treatment strategy based on the degree of response obtained. DWI has been used for providing an early prediction of tumor response in patients who underwent neoadjuvant chemotherapy [21, 22].

Evaluation of residual cancer if any after treatment is important for determining the prognosis of patients, and based on that, management of clinical/surgical treatment modalities could be done. Therefore, keeping in view the merits of DCE- and DWI-MRI in diagnostics, the present study was performed to evaluate the role of functional and dynamic MRI in the assessment of breast lesions following BCS in TNBC subtypes.

## 2. Patients and Methods

This prospective study involved 50 patients with early-stage TNBC according to TNM staging 8^th^ edition [23]. All patients were evaluated after the end of treatment, which consisted of 3D conformal radiotherapy (whole breast radiotherapy 4240 cGy/16 fx − 265 cGy/fx with electron boost 1000 cGy/5 fx − 200 cGy/fx), and in case of chemotherapy, it consisted of 4 cycles of AC, followed by 12 weeks of paclitaxel. During the follow-up period, when a suspicious mass was detected by ultrasonography or mammography, multiparametric MRI was done. Patients were referred from the oncology department to the radiodiagnosis department, Tanta University Hospital, during the period of April 2017 to April 2020.

Breast lesions detected during sonomammography examinations that scored from BIRADS 3 to 5 according to the BIRADS classification were included in the analysis [24].

Pregnant women; patients with chronic renal impairment, previous allergies to contrast, and implantable devices that are not MRI-compatible; and those with particularly large breasts were excluded from the study. Patients were aware of the examination, and informed consent was obtained prior to the analysis. Ethics approval was acquired from the institutional ethical committee.

Complete history of patients was obtained, including personal details, menstrual cycle, and complete clinicopathological data, i.e., details of oncological treatment (e.g., radiotherapy and chemotherapy). TNBC was defined as the lack of estrogen, progesterone, and her2 receptor staining by immunohistochemistry or gene amplification using fluorescent in situ hybridization according to the ASCO/CAP guidelines [25]. The conducted metastatic workup included chest X-ray, abdominopelvic ultrasonography, bone scan, and computed tomography (CT) scan of the chest or abdominopelvis with contrast, if indicated. Sonomammography was performed for all patients. Suspicious lesions on the surgical bed were scored from BIRADS 3 to 5. Subsequently, MRI was conducted to examine the suspicious lesions.

### 2.1. MRI Technique

A closed high-speed MRI machine (General Electric SIGNA 1.5 T) equipped with bilateral breast coils was used for 50 patients. For premenopausal patients, examination was conducted on days 6–13 of the menstrual cycle. Transverse, sagittal, and coronal plane localization scans were subsequently done. Patients were examined by fast spin echo (FSE) T1WI (*T*_*R*_ 8.6 ms, *T*_*E*_ 4.7 ms), T2WI with short tau inversion recovery (STIR) (*T*_*R*_ 5600 ms, *T*_*E*_ 59 ms), and DWI in transverse plan using a single-excitation echo planar imaging sequence (*T*_*R*_ 8400 ms, *T*_*E*_ 98 ms).

Dynamic contrast MRI was performed by two-dimensional fast spoiled gradient recalled echo with fat suppression in T1WI (*T*_*R*_ 4.3 ms, *T*_*E*_ 1.3 ms). Five-phase dynamic images were acquired at 1,2, 3, 4, and 5 min. Dynamic analysis with generation of the percent of enhancement vs. time curves was performed by positioning the area of interest for all identified lesions with a diameter greater than 5 mm.

Lesions showing enhancement were assessed for the pattern of enhancement, i.e., rim-, mass-, and non-mass-like enhancement. The diameter of the region of interest (ROI) was 5–25 mm^2^. A small ROI was allowed near the tumor edge to achieve greatest accuracy. ROI was placed over the enhancing lesion to obtain a dynamic curve pattern. The initial phase of enhancement occurred within 10 min of the contrast injection. The delayed phase was described as the persistent, plateau, or washout phase.

Subsequently, a visual analysis of the diffusion-weighted images was conducted and the apparent diffusion coefficient (ADC) measurement was performed. Images were classified based on the acquired diffusion images and ADC value. High-diffusion images and low ADC value were conducive to restricted diffusion. Moderate signal intensity in both diffusion images and ADC map were conducive to nonrestricted diffusion.

ADC values were identified from b-800 DWI-MRI. If identification based on b-800 DWI-MRI was not possible, the lesion was evaluated by b-50 or b-400 images.

The enhancement percentages as well as the shapes of the curves (type I, II, and III curves) were examined. The breast imaging reporting and data system (BIRADS) was used in the study [26].

Heterogeneous mass or nonmass enhancement with ill-defined or irregular margins and observation of a type 3 curve in dynamic analysis were consistent with a malignant lesion diagnosis (BIRADS 4 and 5). Observation of a well-defined regular nonenhancing mass and type 1 curve in dynamic analysis were conducive to a benign lesion diagnosis (BIRADS 2).

No definite diagnosis was achieved for cases in between these two types and those exhibiting type 2 curves. Hence, further assessment by biopsy and follow-up was recommended (BIRADS 3).

### 2.2. Pathological Assessment

Patients were assessed using Tru-cut biopsy to define the nature of suspicious lesion.

### 2.3. Statistical Analysis

Data was analyzed in terms of range and mean ± standard deviation. Student's *t*-test was used for comparison of the data. Further, *p* values of <0.05 were considered statistically significant. Accuracy, sensitivity, specificity, positive predictive value, and negative predictive value were determined.

A true positive was defined as BI-RADS ≥ 4 and proven as recurrence on pathological evaluation, while a false positive was defined as the same BI-RADS but proven to be a benign lesion on pathological evaluation.

A false negative was defined as BI-RADS ≤ 3 and proven as recurrence on histopathology, while a true negative was defined as the same BI-RADS but proven as a benign lesion on histopathology.

## 3. Results

In this study, 43 cases (86%) were found to be premenopausal. Invasive ductal carcinoma was histopathologically diagnosed in 20 cases (40%). Most of the patients were treated by BCS with postoperative radiotherapy. Only 47 patients (90%) received chemotherapy, and 34 cases (64%) were classed as BIRADS 4 and 5 based on the sonomammography data. Final histopathological diagnosis of recurrence was reported in 10 patients (20%) (Table 1).

On analyzing the morphology of lesions, 20 cases (40%) had regular lesions, while 28 cases (56%) had irregular changes were noted. Moreover, in 2 cases (4%), non-mass-like patterns were observed. Well-defined margins were detected in 20 cases. Twelve cases exhibited nonenhancement. Enhanced lesions were determined in 38 patients. Among them, 18 (36%), 15 (30%), and 5 lesions (10%) displayed rim, heterogeneous, and homogenous enhancements, respectively. For the 38 cases exhibiting enhancement, type I, II, and III curves were noted in 44%, 14%, and 18% of cases, respectively (Table 2).

According to the visual analysis of signal intensity in the diffusion sequence performed at different *b* values, 15 cases (30%) showed restricted diffusion with ADC values in the range of 0.72–1.09 (×10^−3^mm^2^/s) with a mean of 0.88 ± 0.13.

No diffusion restriction was observed in 35 cases (30%) with an ADC value in the range of 1.22–1.81 (×10^−3^mm^2^/s) with a mean of 1.38 ± 0.18 (×10^−3^mm^2^/s).

Final histopathological diagnosis of neoplastic recurrence was made in 10 cases. Among them, 8 cases (16%) were determined as invasive ductal carcinomas and 2 cases (4%) as invasive lobular carcinomas. Forty cases were negative for malignancy with 21 (42%) and 19 cases (38%), as fat necrosis and fibrosis, respectively. Mean ADC for benign postoperative lesions was calculated to be 1.40 ± 0.20 × 10^−3^mm^2^/s and was significantly higher than that for malignant tumors (0.89 + 0.14 × 10^−3^mm^2^/s) (*p* value of <0.001).

Overall MRI examination revealed 11 (22%) suspicious lesions and 39 (78%) nonsuspicious lesions. Final MRI diagnoses made in 11 (22%), 20 (40%), and 19 cases (38%) were recurrence, fat necrosis, and fibrosis, respectively (Table 3). MRI diagnosis was subsequently correlated with histopathological diagnosis. It was found that one false-positive case was histopathologically diagnosed as fat necrosis.

Furthermore, MRI evaluation of the breast lesions following the BCS treatment showed 100% sensitivity, 92.5% specificity, 90.9% positive predictive values, 100% negative predictive values, and 98% accuracy.

### 3.1. Case 1

**Figure 1:**
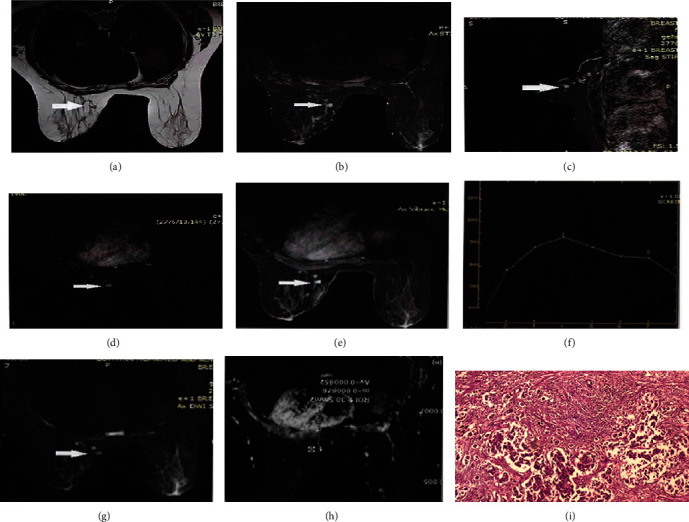
(a) T2WI 2 small lesions at the upper inner quadrant of the left breast with low signal intensity. (b, c) Axial and sagittal STIR sequence lesions showed high signal intensity with no evidence of fat expression. (d) Subtraction images: enhancement of lesions. (e) Post contrast T1 WI homogenous enhancement of operative lesions. (f) Time intensity showed curve type III. (g) High signal intensity was seen in DWI. (h) Low signal on ADC map with ADC value = 0.9 × 10^−3^ mm^2^/s. MRI diagnosis was recurrence, consistent with histopathology. (i) Histopathology recurrent infiltrating duct carcinoma.

A premenopausal patient with triple-negative left breast cancer (T2N1M0) diagnosis was treated with BCS, followed by chemotherapy and radiotherapy for 18 months. New breast ultrasonography revealed multifocal hypoechoic lesions with irregular outlines, which were highly suspicious for malignancy. MRI showed multiple enhancing subcentimetric soft tissue lesions in the upper inner quadrant of the left breast at the site of previous operation. Kinetic assessment demonstrated a type III washout curve with restricted diffusion, denoting malignant nature of the lesions (BIRADS 5). This indicated recurrence, which was confirmed further by histopathology (Figure 1).

### 3.2. Case 2

**Figure 2:**
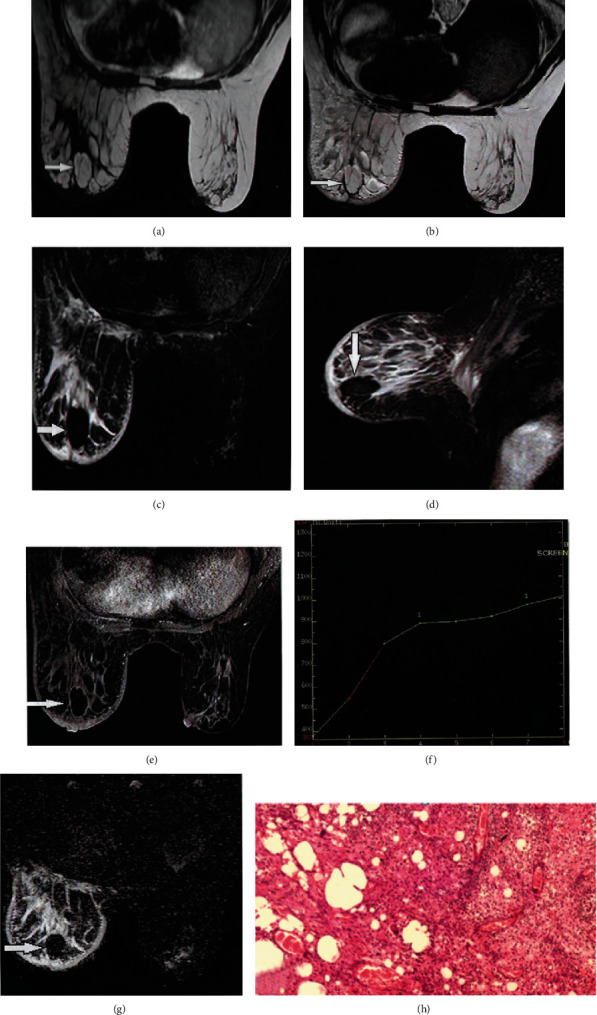
(a, b) Axial T1WI and T2WI showed well-defined isointense lesion at the upper outer quadrant of the left breast. (c, d) Axial and sagittal STIR demonstrated loss of signal. (e) Axial T1WI post contrast showed marginal faint enhancement. (f) Kinetic enhancement showed a type I curve. (g) DWI showed a hypointense signal with an ADC value 1.42 × 10^−3^ mm^2^/s consistent with fat necrosis. (h) Histopathology revealed fat necrosis.

A postmenopausal patient with triple-negative breast cancer (T1N0M0, invasive ductal carcinoma) was treated with BCS, followed by radiotherapy two years ago. During follow-up, ultrasonography revealed an irregular area at the upper outer quadrant of the left breast (BIRADS 3). MRI showed an irregular area at the upper outer quadrant of the left breast at the operative bed, while the kinetic assessment demonstrated a type I curve, denoting a benign nature of the lesion (BIRADS 3). The final MRI diagnosis was fat necrosis, which was consistent with histopathological examination (Figures 2(a) and 2(b)).

### 3.3. Case 3

**Figure 3:**
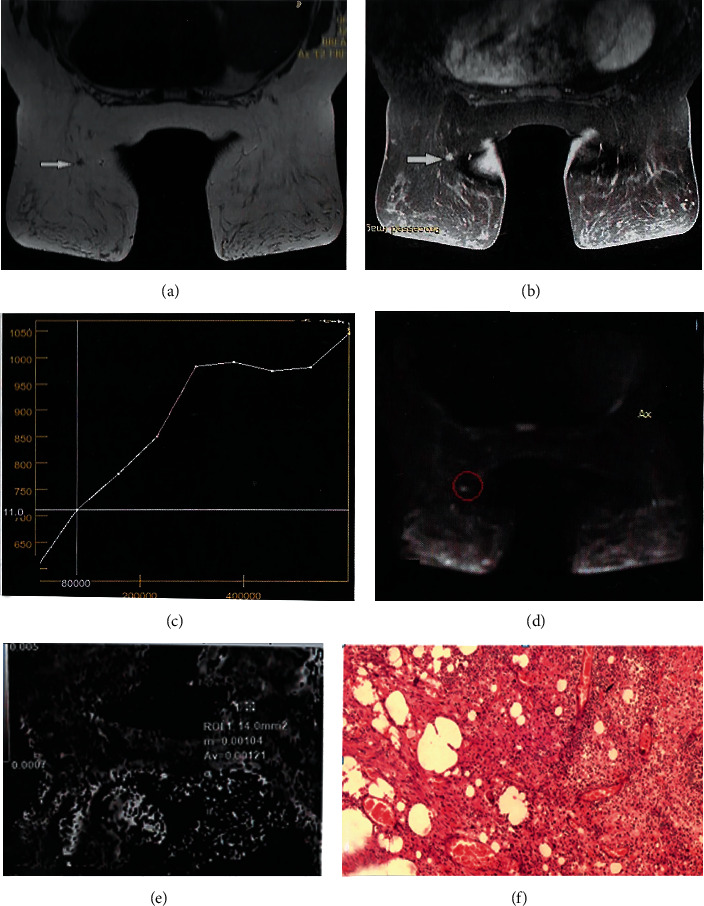
(a) Axial T3 WI irregular area of low signal intensity at the lower inner quadrant of the left breast. (b) Axial T1WI post contrast showed enhancement at the area of interest. (c) Kinetic assessment of the enhanced area showing a rising curve denoting benign nature. (d) DWI hyper intense signal. (e) ADC map hyper intense signal with a DC value of 12 × 10^−3^ mm^2^/s. MRI diagnosis was postoperative scar, consistent with histopathological diagnosis. (f) Histopathological finding of fibrosis.

A postmenopausal patient with triple-negative breast cancer (T1N0M0, invasive ductal carcinoma) was treated with BCS, followed by radiotherapy. During follow-up, ultrasonography revealed an irregular area at the lower inner quadrant of the left breast (BIRADS 3). MRI examination showed an irregular area at the lower inner quadrant of the left breast at the operative bed, while the kinetic assessment demonstrated a type I curve, denoting a benign nature of the lesion (BIRADS 3). The final MRI diagnosis determined postoperative fibrosis, which was in agreement with the histopathological assessment (Figures 3(a) and 3(b)).

## 4. Discussion

BCS has been increasingly integrated into breast cancer management [27]. It is the standard and safe therapeutic procedure for the early stage of the disease. BCS involves lumpectomy or quadrantectomy with/without axillary lymph node dissection. It is typically followed by breast radiotherapy and results in survival rates comparable to those observed after mastectomy. Although the recurrence rate is low, it is not nonexistent [28, 29].

Local recurrence rates were determined at 2–3% and 10–12% following BCS or mastectomy in patients with luminal A subtype and triple-negative cancers [1]. Compared with other breast cancer subtypes, majority of recurrence in TNBC occurred during the first 5 years [30, 31].

Typically, tumors recur at the lumpectomy bed, adjacent to the margin, or elsewhere in the breast [32–35]. Mammography and breast ultrasonography failed to establish the nature of recurrence due to architectural distortion, increased density at the lumpectomy site, and posttreatment edema [36–38]. Dynamic breast MRI is considered as a valuable technique for patients with suspected recurrence after BCS [36]. Sensitivity of breast MRI for evaluation of recurrence has been reported at 90% [35, 39]. Diffusion-weighted MRI exhibits improved specificity and positive predictive value compared to conventional MRI [40, 41].

The aim of the present study was at examining the role of dynamic and functional MRI in evaluating breast cancer recurrence in patients with TNBC to further determine whether recurrence was benign or malignant.

This prospective study involved 50 patients with early-stage TNBC who underwent BCS. All patients received radiotherapy, and 47 of them were also treated with chemotherapy. Suspected breast lesions were evaluated by sonomammography. Metastatic workup was performed to exclude distant metastases. Subsequently, functional and dynamic MRI was conducted.

Recurrence at the operative bed was suspected in all 50 cases based on the sonomammography assessment. Eighteen lesions were classed as BIRADS 3 (36%), 26 (52%) as BIRADS 4, and 6 (12%) lesions as BIRADS 5. All patients were referred for an MRI of the breast to verify the nature of the suspicious lesions.

As previously reported by Kilic et al., premenopausal patients underwent MRI on days 6–13 of their menstrual cycle to reduce the risk of false positives [42].

Suspicious morphological criteria (i.e., irregular and speculated outline) were found in 20 lesions (40%). Thirty lesions (60%) in our study exhibited benign morphological features such as a well-defined margin and smooth outline, which were in agreement with previous reports [43, 44].

Thirty-eight cases showed enhancement in the MRI study. The investigation of the enhancement using a kinetic MRI study revealed a type I curve in 22 lesions (44%). Additionally, a type II plateau curve was noted in 7 lesions (14%) and a type III washout curve was observed in 9 lesions (18%). Curves indicated the presence of benign, suspicious, or malignant lesions, respectively [45, 46].

According to the results of DCE-MRI, 12 out of 50 studied lesions were suspected for malignancy. Among them, 10 lesions (20%) were pathologically confirmed as malignant, with a positive predictive value of 83.3%. Two cases exhibited a type II curve, while eight displayed a type III curve. Two of the 50 cases (4%) were upgraded to BIRADS 4 based on DCE-MRI; however, they were subsequently determined as fat necrosis by histopathological analysis [36, 39].

Diffusion-weighted images were obtained prior to contrast enhancement to avoid interference by the contrast material. Diffusion analysis was performed by visual assessment of the lesion using different *b* values. According to the previous studies, the cutoff value for ADC between benign and malignant lesions was established at 1.09 × 10^−3^ mm^2^/s [47].

Fifteen cases (30%) showed restricted diffusion with ADC values in the range of 0.72 ± 1.09 (×10^−3^mm^2^/s), with a mean of 0.88 ± 0.13. No diffusion restriction was reported in 35 cases (30%) with ADC ranging from 1.22 to 1.81 (×10^−3^mm^2^/s), with a mean of 1.38 ± 0.18 (×10^−3^mm^2^/s). These outcomes were consistent with the previously reported results [48, 49].

Compared to locoregional recurrence, all cases of postoperative scars studied in this work displayed high mean ADC values [47–49].

Overall, in the present study, the final MRI diagnosis correlated with the histopathological diagnosis. There was one false-positive case, which was histopathologically diagnosed as fat necrosis [50]. Combining the DWI-MRI and DCE-MRI data resulted in 100% sensitivity, 92.5% specificity, 90.9% positive predictive value, 100% negative predictive value, and 98% accuracy [50, 51].

Based on the observations made in this study, it was concluded that the assessment of breast lesion reoccurrence in TNBC after BCS and radiotherapy with or without chemotherapy using dynamic and functional MRI exhibited a high negative predictive value with no false-negative cases. These results were further confirmed by histopathological examination.

Thus, the combination of dynamic and functional MRI could serve as a suitable technique for accurate detection and evaluation of early lesions in the breast.

## Figures and Tables

**Table 1:** Patients' characteristics of fifty patients.

Pathology		
Invasive ductal carcinoma	20	40
Invasive lobular carcinoma	16	32
Mixed ductal and lobular carcinoma	14	28
Treatment received		
Conservative breast surgery	50	100
Radiotherapy	50	100
Chemotherapy	47	94
BI-RADS lexicon		
BI-RADS 3	18	36
BI-RADS 4	26	52
BI-RADS 5	6	12
Final histopathological diagnosis		
Recurrence	10	20
Postoperative changes	40	80

**Table 2:** Type of dynamic curves in the cases of enhancement (38 cases).

Type of dynamic curves	No.	%	Interruption
Type I	22	44	Benign lesions
Type II	7	14	Suspicious lesions
Type II	9	18	Malignant lesions
Total	38	76	

**Table 3:** Correlation between DCE-MRI, diffusion findings, final MRI, and the final results.

	DCE-MRI diagnosis			
	Suspicious (12)		Nonsuspicious (38)	
	No	%	No	%
Histopathological result				
Recurrence (10)	10	83.3	0	0
Postoperative changes (40)	2	16.7	38	100

	DWI-MRI finding			
	Suspicious (12)		Nonsuspicious (39)	
	No	%	No	%
Histopathological result				
Recurrence (10)	10	90.9	0	0
Postoperative changes (40)	1	9.1	39	100

	Final diagnosis			
	Final MRI		Histopathological diagnosis	
	No	%	No	%
Neoplastic recurrence	11	22	10	20
Fat necrosis	20	40	21	42
Scar fibrosis	9	38	19	38

## Data Availability

Data will be available upon request.

## Abbreviations

TNBC: Triple-negative breast cancer
BCS: Breast-conserving surgery
MRI: Magnetic resonance imaging
DCE: Dynamic contrast enhanced
DWI: Diffusion-weighted imaging
ADC: Apparent diffusion coefficient
BIRADS: Breast imaging reporting and data system.
